# Data of characterization of sodium pectate complexes with iron and manganese

**DOI:** 10.1016/j.dib.2021.107594

**Published:** 2021-11-19

**Authors:** Kirill Kholin, Guliya Nizameeva, Salima Minzanova, Marsil Kadirov

**Affiliations:** a FRC Kazan Scientific Center, Arbuzov Institute of Organic and Physical Chemistry, Russian Academy of Sciences, Kazan 420088, Russia; b Kazan National Research Technological University, Kazan 420015, Russia

**Keywords:** Fourier-transform infrared spectroscopy, Electron spin resonance, Electrochemistry, Atomic force microscopy

## Abstract

Data for iron and manganese-containing sodium pectate complexes are reported. Such complexes are potentially capable of exhibiting catalytic properties to the electroreduction of small molecules. Also, the complexes are water-soluble due to their ligands. The combination of these factors makes them promising for homogeneous electrocatalysis. However, in many respects, these complexes remain poorly understood. The Fourier-transform infrared spectroscopy data for the sodium pectate complexes with manganese and iron in the range of 500–4000 cm^−1^ were obtained. The electron spin resonance spectra of the complexes make it possible to characterize oxidation states of the metal centers in the complexes. The cyclic voltammetry data for the complexes both in an aqueous solution saturated with argon and saturated with carbon dioxide were received. For both complexes after deposition of the complexes on graphite managed to get micrographs by the atomic force microscopy method.

**Specifications Table**

**Table:** 

Subject	Inorganic Chemistry; Analytical Chemistry: Spectroscopy; Electrochemistry
Specific subject area	Physicochemical characterization of the sodium pectate complexes with iron and manganese
Type of data	Figure
How the data were acquired	The data were acquired using IR-Fourier spectrophotometer Tensor 27 (Bruker), Electron spin resonance spectrometer of the X-range ELEXSYS E500 (Bruker), Epsilon EClipse electrochemical analyzer (BASi) with C-3 Cell Stand, Atomic force microscope MultiMode V (Veeco)
Data format	Raw
Description of data collection	The infrared and electron spin resonance spectra of the complexes were recorded at room temperature. The pH of aqueous solutions for electrochemistry was maintained at a level of 7,0. The scan rate in electrochemical experiments was 100 mV/s. The scanning rate for AFM measurements was 1 Hz.
Data source location	Institution: Arbuzov Institute of Organic and Physical Chemistry, FRC Kazan Scientific Center, Russian Academy of Sciences City/Town/Region: Kazan Country: Russia
Data accessibility	With the article and in a public repository: Repository name: Mendeley Data Direct URL to data: 10.17632/n92mmn4pxc.1
Related research article	V.A. Milyukov, A.V. Khabibullina, D.M. Arkhipova, V.F. Mironov, A.R. Khamatgalimov, I.S. Ryzhkina, L.I. Murtazina, L.G. Mironova, A.B. Vyshtakalyuk, A.V. Nemtarev, N.G. Nazarov, K.V. Kholin, I.R. Nizameev, S.T. Minzanova, Synthesis, Physicochemical Properties and Anti-Fatigue Effect of Magnesium, Zinc and Chromium Polygalacturonate Based Composition, ChemistrySelect. 4(14) (2019) 4331–4338 [1]. 10.1002/slct.201803812

**Value of the Data**

• It's known that polysaccharides and their derivatives have electrocatalytic properties to the hydrogen evolution reaction [2,3]. The synthesis of metal complexes based on polysaccharides makes it possible to influence these properties [4]. In addition, such complexes sometimes exhibit catalytic activity in other reactions [5]. However, there is still very little data on some of these complexes.
• The data will be useful to a wide range of researchers engaged in homo and heterocatalysis of the hydrogen evolution and carbon dioxide reduction reactions (HER and CO2RR).
• The infrared and electron spin resonance spectra of the sodium pectate complexes with manganese and iron allow to determine the state of COO− groups and metal centers. These data can be used/reused for further insights of the structure and properties of the complexes.
• Cyclic voltammetry of the complexes in aqueous solutions saturated with argon or carbon dioxide demonstrates the electrocatalytic response of the complexes to HER and/or CO2RR and allows one to determine onset potentials of these processes.
• Very large sizes of the complexes and the presence of many metal centers determine their pseudo-heterogeneous behavior under homogeneous conditions. The data of atomic force microscopy of the graphite surface with complexes deposited on it can be used/reused to determine the size of molecules and the nature of their interaction with the surface.

## 1 Data Description

All experiments were carried out with the sodium pectate complexes of iron (PG-NaFe) and manganese (PG-NaMn). The complexes have been obtained by 20% replacement of sodium ions in sodium pectate with iron or manganese ions, respectively. This ratio was selected experimentally. At a higher degree of ion substitution, the complexes became water-insoluble.

**Fig. 1:**
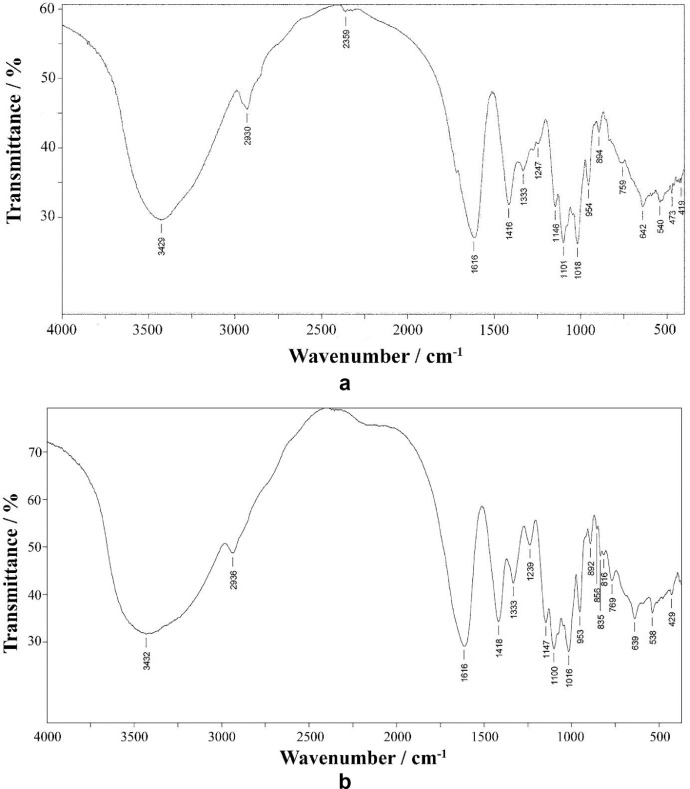
Fourier-transform infrared spectra of the sodium pectate complexes with (**a**) iron (PG-NaFe) and (**b**) manganese (PG-NaMn).

**Fig. 2:**
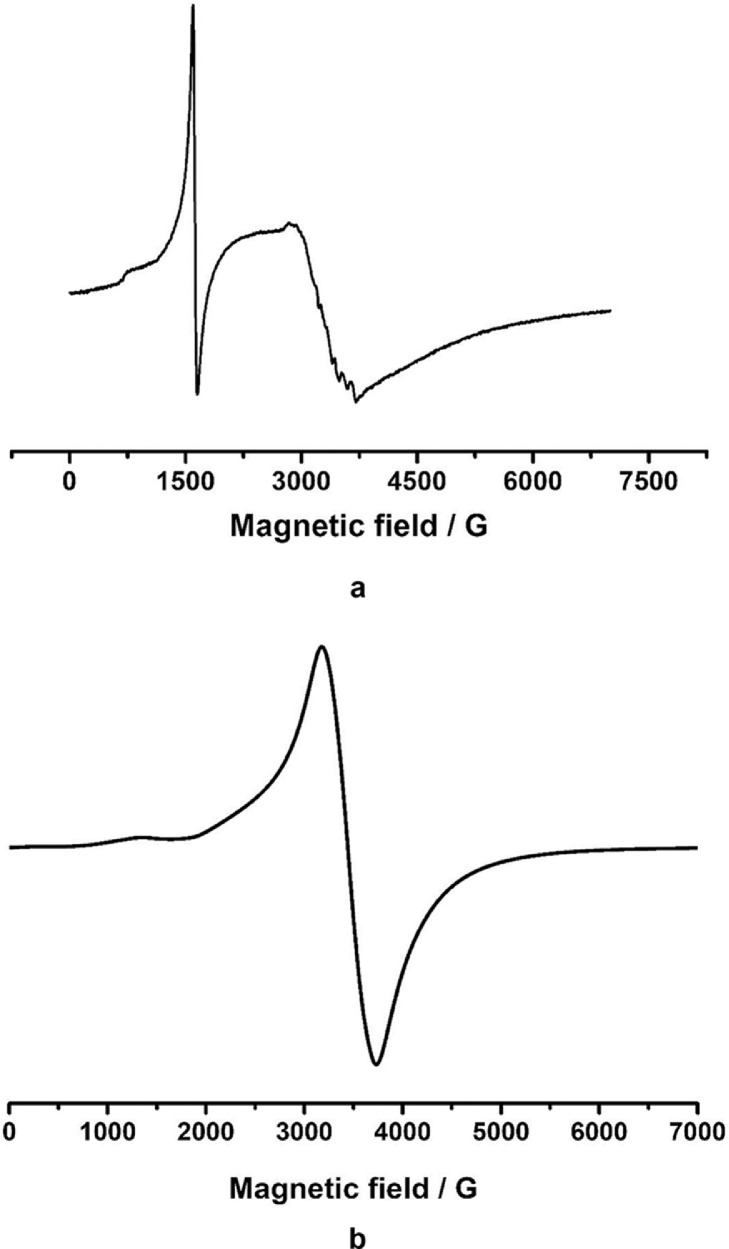
Electron spin resonance spectra of the sodium pectate complexes with (**a**) iron (PG-NaFe) and (**b**) manganese (PG-NaMn) in solid powder state.

**Fig. 3:**
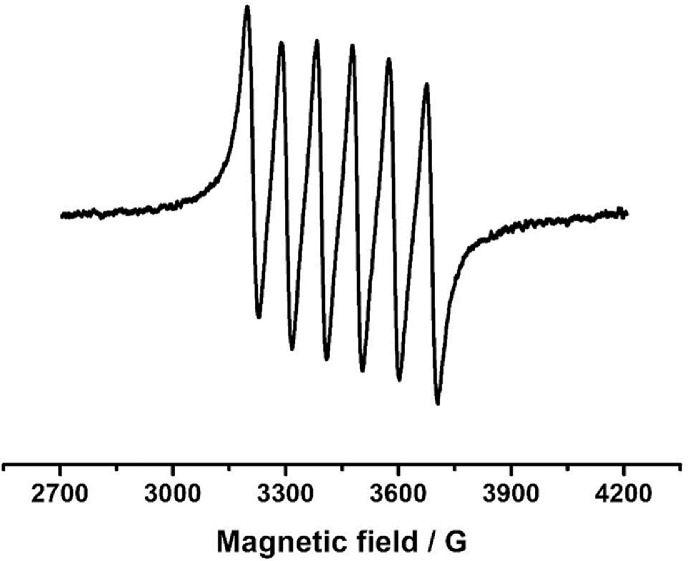
Electron spin resonance spectrum of the sodium pectate complexes with manganese (PG-NaMn) in in aqueous solution.

**Fig. 4:**
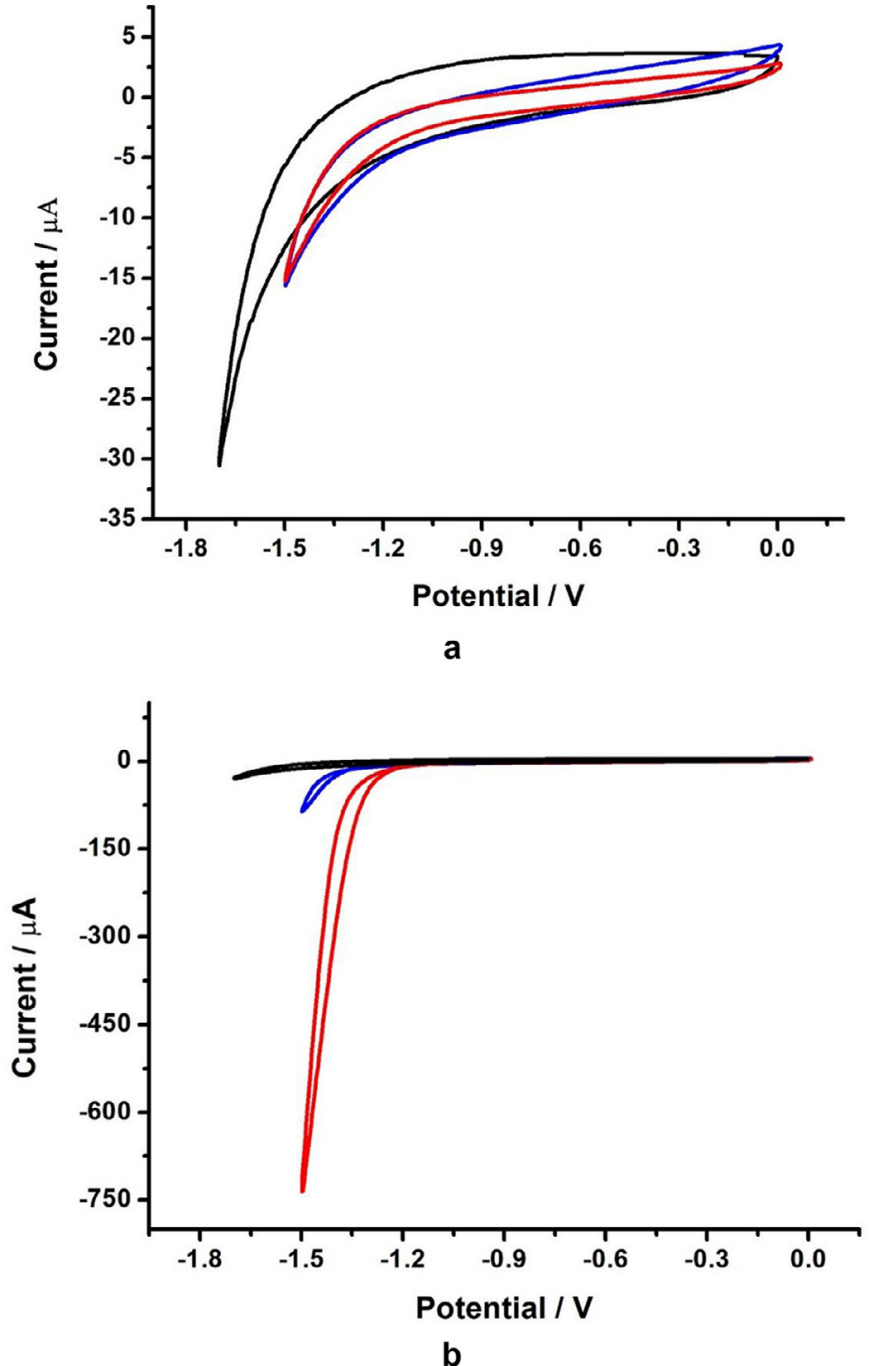
Сyclic voltammograms recorded at a glassy carbon electrode (GCE) in the presence of the (**a**) PG-NaFe and (**b**) PG-NaMn complexes in aqueous solutions saturated with argon (blue curves) or carbon dioxide (red curves). Black lines are background in the absence of the complexes.

**Fig. 5:**
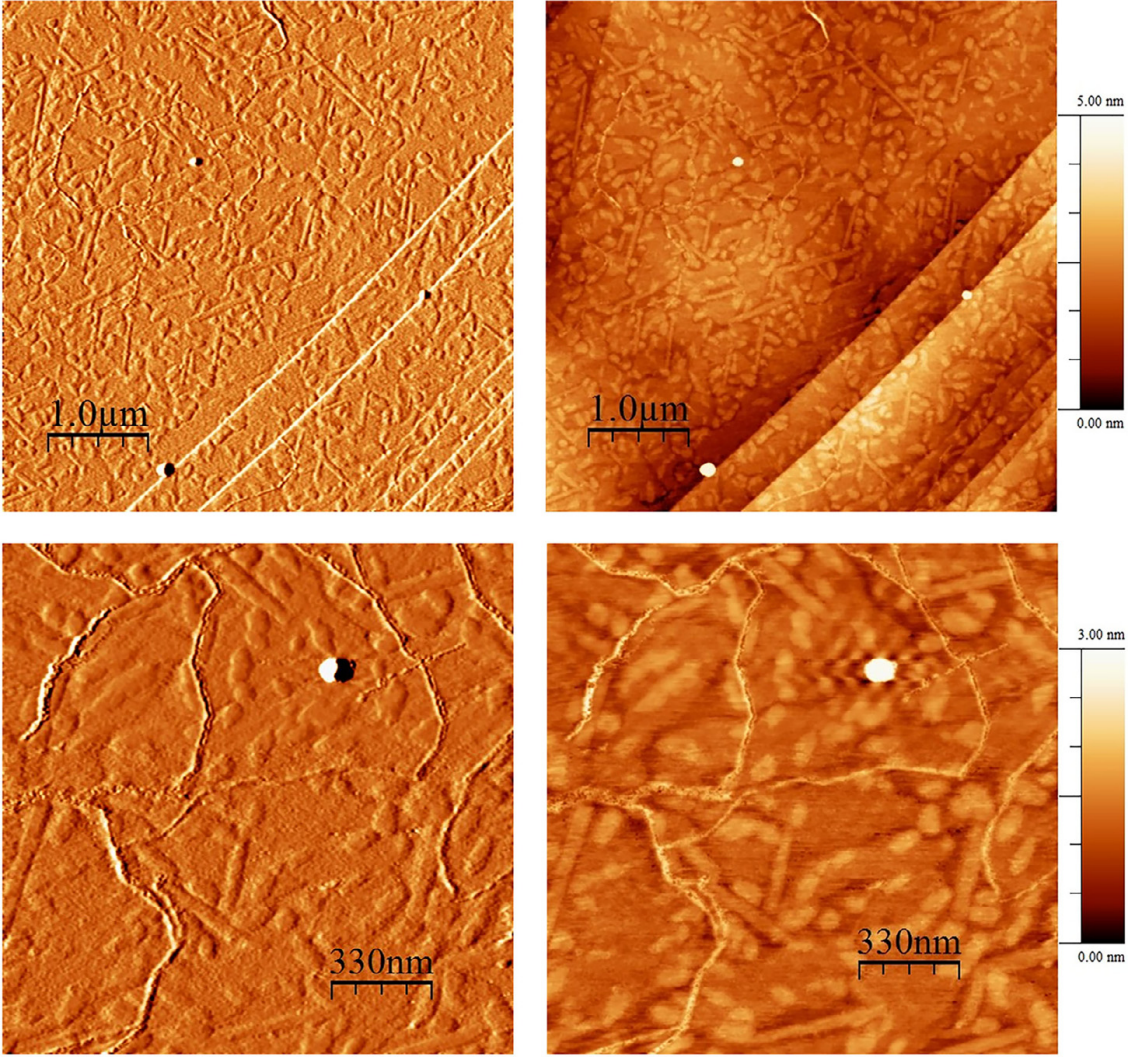
Atomic force microscopy images of graphite surface with the deposited iron complex (PG-NaFe).

**Fig. 6:**
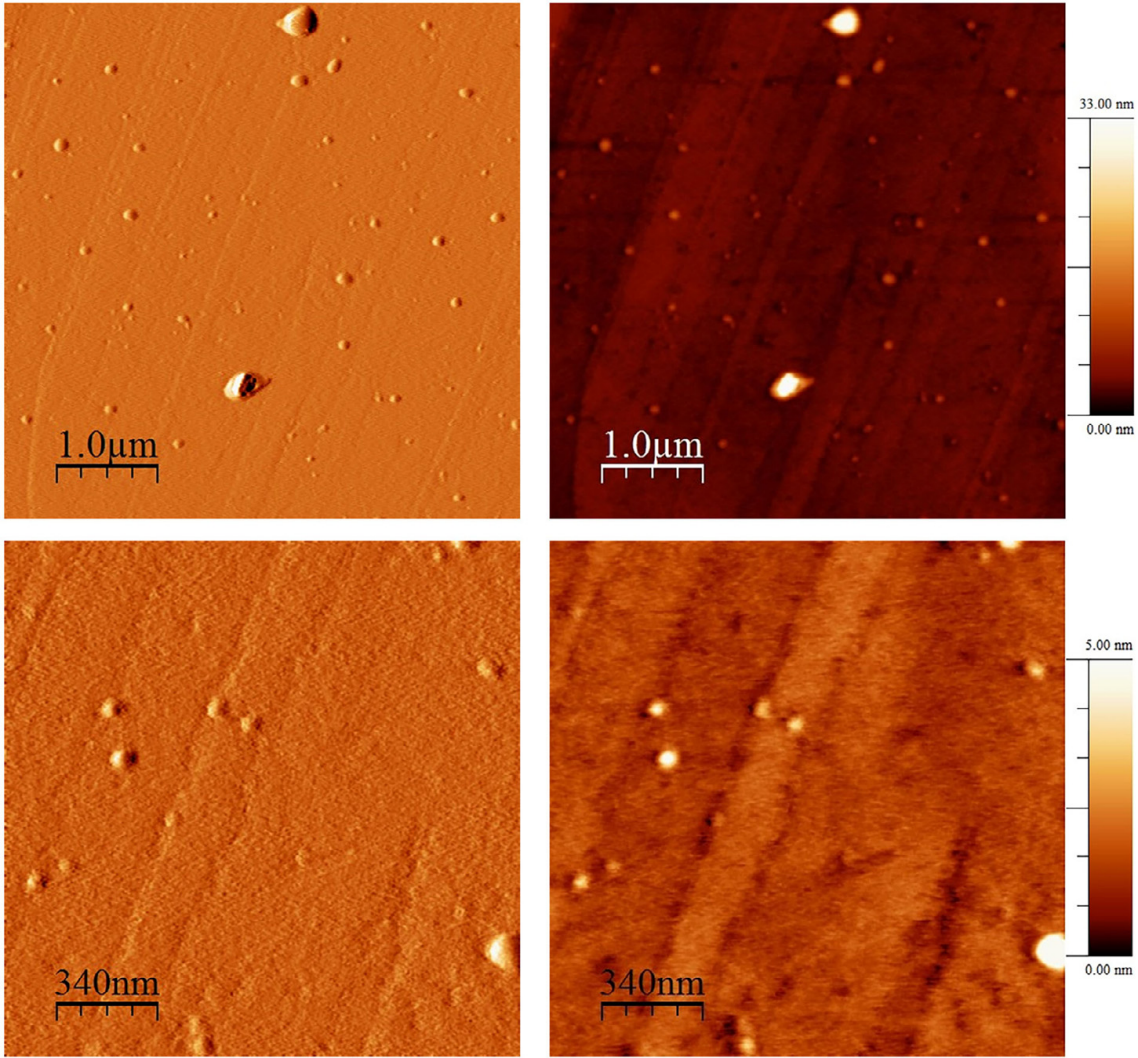
Atomic force microscopy images of graphite surface with the deposited manganese complex (PG-NaMn).

Fourier-transform infrared analysis of the PG-NaFe (Fig. 1a) and PG-NaMn (Fig. 1b) complexes was performed. These transmission spectra provide the data on the vibrations of bonds and atomic groups in the complexes. In particular, both spectra have characteristic absorption bands at 1616 cm^−1^ corresponding to valence vibrations of COO^−^ group and there are no absorption bands at 1745–1750 cm^−1^ corresponding to C=O group valence vibrations. The powders of the complexes have electron spin resonance (ESR) spectra, as can be seen from Fig. 2. In both cases, both signals with g-factors close to 2 and signals in a half-field are observed. Integral intensity of the spectrum of the iron complex is about 104 times less than that of the PG-NaMn complex. The electron spin resonance spectrum of the PG-NaMn complex in an aqueous solution is shown in Fig. 3. In this case, one can observe the hyperfine structure with hyperfine constant a_Mn_ = 95 G. Aqueous solution of the PG-NaFe complex does not have an ESR spectrum and, therefore, there is no corresponding figure. Cyclic voltammetry curves of the complexes on a glassy carbon electrode in an aqueous solution are shown in Fig. 4. The iron complex in solutions saturated with argon and carbon dioxide gives close values of onset potential E_onset_. In addition, the E_onset_ in these cases is only slightly more positive than in an aqueous solution without the complex. The manganese complex behaves completely differently. E_onset_ for PG-NaMn complex solution saturated with argon is more positive than E_onset_ for solution without the complex by 150 mV. And when the solution is saturated with carbon dioxide, the E_onset_ shifts by an additional 150 mV. The data on the surface morphology after the deposition of the complexes on graphite were obtained by atomic force microscopy (Fig. 5 for PG-NaFe and Fig. 6 for PG-NaMn). In the case of the iron complex, we see formations with lateral dimensions of hundreds of nanometers, while the manganese complex forms nanoparticles with tens of nanometers in size.

## 2 Experimental Design, Materials and Methods

Synthesis of the sodium pectate complexes was carried out using a known method for water-soluble complexes of pectin polysaccharides obtaining [6]. Citrus pectin “Classic C-401” produced by Herbstreith & Fox and metal salts FeSO_4_ and MnSO_4_ with a purity of more than 99.9% were used. Deionized water used as a solvent in the synthesis. Preliminarily distilled water was passed through a Simplicity water purification system from Merck Millipore to obtain deionized water. Water was considered suitable for conducting experiments with a resistivity factor of more than 18.2 MΩ•cm at 25 °C. The same water was used as a solvent in ESR and electrochemical experiments. IR spectra were recorded on IR-Fourier spectrophotometer Tensor 27 (Bruker) with 1 cm^−1^ resolution in the range 400–4000 cm^−1^, the substance being pressed with KBr in tablets.

Electron spin resonance spectra were obtained using a spectrometer ELEXSYS E500 of the X-range. Samples for ESR experiments were placed in quartz ampoules. Aqueous samples were placed in thin ampoules with an inner diameter of 1 mm. BASi Epsilon EClipse electrochemical analyzer (USA) was used for electrochemistry. A conventional three-electrode system [7] was used with glassy carbon as the working electrode (1.5 mm diameter), the Ag/AgCl (3 M) electrode as the reference electrode, and a Pt wire as the counter electrode. The pH of aqueous solutions for electrochemistry was maintained at a level of 7,0 with sodium phosphate buffer. An atomic force microscope (MultiMode V, USA) was used to reveal the morphology of graphite surfaces with the complexes. The 250–350 kHz cantilevers (Veeco, USA) with silicone tips (tip curvature radius is 10–13 nm) were used in all measurements. The microscopic images were obtained with 512 × 512 resolution. The scanning rate was 1 Hz. The antivibrational system (SG0508) was used to eliminate external distortions. Droplets of dispersions of the sodium pectate complexes were carefully placed on a graphite surface with a roughness no more than 1 nm. The AFM imaging was performed after solution evaporation.

## Ethics Statements

The authors declare that this work does not involve any data with the use of human subjects or animal experiments and does not involve data collected from social media platforms.

## CRediT Author Statement

**Kirill Kholin:** Conceptualization, Methodology, Resources, Supervision, Investigation, Validation, Writing – original draft, Visualization. **Guliya Nizameeva:** Investigation, Validation, Visualization. **Salima Minzanova:** Investigation, Validation, Visualization. **Marsil Kadirov:** Investigation, Validation.

## Declaration of Competing Interest

The authors declare that they have no known competing financial interests or personal relationships that could have appeared to influence the work reported in this paper.
